# Ultracompact CMOS-compatible optical logic using carrier depletion in microdisk resonators

**DOI:** 10.1038/s41598-017-12680-1

**Published:** 2017-10-03

**Authors:** Dusan Gostimirovic, Winnie N. Ye

**Affiliations:** Carleton University, Silicon Micro/NanoPhotonics Group, Ottawa, K1S 5B6 Canada

## Abstract

We present a CMOS-compatible optoelectronic directed logic architecture that achieves high computational throughput (number of operations per second per unit area) by its ultracompact form factor. High speed-to-power performance is also achieved, by the low capacitance and high junction-to-mode overlap of low-radii SOI vertical pn junction microdisk switches. By using wavelength-division multiplexing and two electrical control signals per disk, each switch performs (N)OR, (N)AND, and X(N)OR operations simultaneously. Connecting multiple switches together, we demonstrate higher-order scalability in five fundamental *N*-bit logic circuits: AND/OR gates, adders, comparators, encoders, and decoders. To the best of our knowledge, these circuits achieve the lowest footprint of silicon-based multigigabit-per-second optical logic devices in literature.

## Introduction

The billions of underlying nanoscale transistors driving modern high-performance computers have been optimized—through decades of substantial and incremental improvements—to subpicosecond switching speeds and subfemtojoule levels of energy consumption per bit^1^. These metrics are now comparable to the metal interconnects that the processed data is transferred through. Though CMOS transistor technology has begun to plateau in its growth^2^, any further improvements will be bottlenecked by the limits of electronic data transport^3^. Because of this, interest has grown in high-bandwidth and low-power silicon-photonic interconnects for chip-to-chip and on-chip applications^4–6^. With processing itself, however, the current thought is that silicon-photonic technologies cannot compete with CMOS as they do with copper^7^. Although silicon-photonic devices seamlessly integrate with current low-cost and high-volume fabrication processes^8–10^, and offer a potential for ultrahigh bit rates^11,12^, they require relatively high threshold optical nonlinearities for switching^13^, and are comparatively larger in size because of the micron-range wavelength of the guided light. However, a low-level comparison between optical and conventional logic does not take interconnect overhead into account. As high-bandwidth photonic interconnects start to replace copper at the chip level, the necessary optical-electrical-optical (OEO) converters will begin to limit performance by increasing delay, energy consumption, complexity, and footprint. Our architecture provides a direct interface of optics and electronics by combining them at the logic level. This reduces the bottleneck of OEO conversions, while combining the best of electrical control and optical data transport.

We consider the following metrics to be of the most importance to a logic architecture: speed, power consumption, size, and ease of fabrication. Ease of fabrication is best satisfied by using CMOS-compatible materials, like the silicon-on-insulator (SOI) platform, and by using structures and designs that are easily fabricated for high degrees of on-chip planar integration. Though well-performing optical logic has been reported in III-V based semiconductor optical amplifiers (SOAs)^14,15^, their incompatibility with low-cost and high-density (CMOS) manufacturing puts them at a significant disadvantage. Thus, we only considered silicon-photonic devices for this work. The remaining metrics are generally determined by the photonic structure and modulation method implemented in the switch. High-speed logic using cross-phase modulation (XPM)^16^ and four-wave mixing (FWM)^17^ in SOI waveguides require high milliwatt-scale pumping powers or (more often) millimeter-scale path lengths for switching to take effect. Microring/disk resonators keep the same effective path length while taking up significantly less space (low area)^18–21^. High-contrast modulation is achieved in these microresonators by shifting their transmission spectra in relation to the propagating light, through electrically induced free-carrier dispersion (FCD) (by injection^22,23^ or depletion^24–26^), two-photon absorption (TPA) based carrier dispersion^27,28^, thermal effects^29^, and the Kerr effect^30^. We avoid the use of thermal effects and free-carrier injection (optically or electrically induced), as their switching speeds are limited by the microsecond-range thermal response time and the nanosecond-range free-carrier lifetime in SOI waveguides, respectively. The Kerr effect is instantaneous; however, the relatively small third-order nonlinearity in silicon causes picojoule levels of required energy consumption per switch. We find that free-carrier depletion—specifically in vertical pn junction microdisk switches—offers the most optimized performance, with multigigabit-per-second switching speeds and subfemtojoule-per-bit operation being shown in literature. In our opinion, carrier depletion based microresonator switches are the only devices suitable for large-scale integrated optical logic.

Though free-carrier effects in SOI microresonators have already been used to perform logic^31–37^, there is much room for improvement in both the architecture of the logic and the optimization of the switching structure itself. In this work, vertical junction microdisks are used rather than lateral junction microrings, as the same change in depletion width spans a larger percentage of the waveguide confined mode, thereby reducing the required control voltage. This junction is split in half and electrically isolated to create two independently working diodes connected to the two control inputs of a basic logic operation. At the very least, this halves the size of the operation (doubling throughput), as all previous designs use one switch per control input. Furthermore, wavelength-division multiplexing (WDM) enables parallel operations for enhanced functionality per unit area and time or multiple instruction, single data (MISD) processing. With three equally spaced wavelengths, one microdisk switch simultaneously performs OR, AND, and XOR operations—as well as their inversions. These six operations, and the built-in half adder via the XOR and AND operations, make up the fundamental logic cell of our architecture. This all-gate cell is given in comparison to previous demonstrations of CMOS-compatible optical logic that use one or (more often) more switches for each gate operation. A summary of different devices is shown in Table 1. Note that the area values are based on simple calculations of waveguide and resonator dimensions, and the space in between each structure is not accounted for. These values can potentially be reduced further by using lower-radii microresonators. Care must also be taken in comparing the ideal energy and speed figures of simulations with experimental works, as certain factors have not been accounted for (e.g., RC and propagation delays). With all factors considered, our proposed cell still improves computational throughput and efficiency over previous demonstrations of optical logic through compact cell- and circuit-level designs and state-of-the-art modulation. Furthermore, the cell can easily expand to perform compact higher-order operations. We present designs for *N*-bit AND/OR gates, adders, comparators, encoders, and decoders that are each, to the best of our knowledge, the smallest reported of their kind in literature. The cells in these circuits follow the directed logic paradigm^38^, like certain previously reported works^31,33,39–41^, in that they each switch independently of each other, simultaneously. Unlike conventional logic, that accumulates delay at each sequential gate, the relative throughput of this type of architecture grows with the size of the circuit.

**Table 1:** Comparison of Previously Reported CMOS-Compatible Optical Logic Devices.

Photonic Structure	Energy (fJ/bit)	Speed (Gbps)	Area (μm^2^)	Demonstrated Logic Function(s)	Required # of Devices
TPA Waveguide^37^	2 × 10^3^	10	4.8 × 10^3^	NOR	1
Thermal Ring^39^	—	20 × 10^−6^	0.9 × 10^6^	X(N)OR	2
TPA Ring^32^	3.2 × 10^3^	0.31	90	(N)AND	1
FCD Ring Depletion^33^	1.2 × 10^3^	3	8 × 10^3^	OR, AND, X(N)OR, A + $\overline{{\rm{B}}}$, A$\overline{{\rm{B}}}$	8
FCD Ring Injection^40^	—	0.1	630	X(N)OR	2
FCD Ring Depletion^41^	—	12.5	630	X(N)OR	2
This Work (Simulation)	12	28	15	(N)OR, (N)AND, X(N)OR	1

## Switching and logic architecture

**Figure 1:**
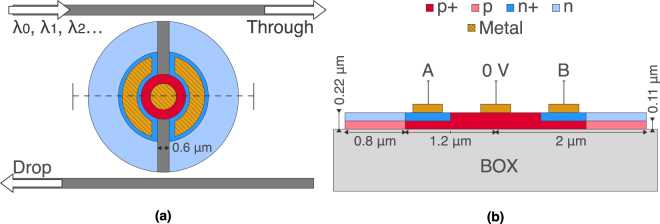
(**a**) Top view of the double-shift vertical pn junction microdisk switch. The gold regions are the metal electrodes; the dark gray regions are of undoped silicon; and the red and blue regions are of p- and n-type silicon, respectively, with darker colors signifying higher concentrations of dopants (p+ and n+). The dashed line is where we take the (**b**) 2D cross section of the device.

The fundamental switch used in our logic architecture is a double-shift vertical pn junction microdisk resonator, as shown in Fig. 1. This resonant photonic device acts as a filter, where most wavelengths of light propagate from the input to the through port, while wavelengths that are integer multiples of the effective path length of the disk cavity exit at the drop port. With small changes to the resulting transmission spectrum—through low-power optically or (in this case) electrically induced adjustments of the refractive index of the cavity—large effective changes are made to where the input light exits. The added doping regions in this disk introduce large concentrations of free holes and electrons that overlap with the whispering gallery mode (WGM). The WGM is a result of total internal reflection of the guided light inside the disk cavity. The extra free carriers reduce the refractive index (blueshifting the transmission spectrum) and increase absorption (reducing the peak depth). Reverse-biasing the pn junction causes an increase in its depletion width; as a result, the WGM overlaps with fewer free carriers, thereby increasing refractive index (redshifting the transmission spectrum back) and decreasing absorption. A key feature of this device is the vertical pn junction, which provides a higher switching efficiency than the conventional lateral junctions used in microring modulators. The change in free-carrier concentration impacts a larger percentage of the vertically confined mode, promoting order-of-magnitude larger wavelength shifts per volt^24^. Furthermore, without the need for a scatter-inducing inner etch, disks confine the propagating mode better, allowing for lower-radii resonators. The p- and n-doped regions of our disk are each easily split to create two independently working diodes connected to two separate electrical control signals. With this, each control has the ability to shift the transmission peak on and off resonance with the propagating optical signal(s), allowing a single switch to produce a 2-bit logical response.

### Switching analysis

**Figure 2:**
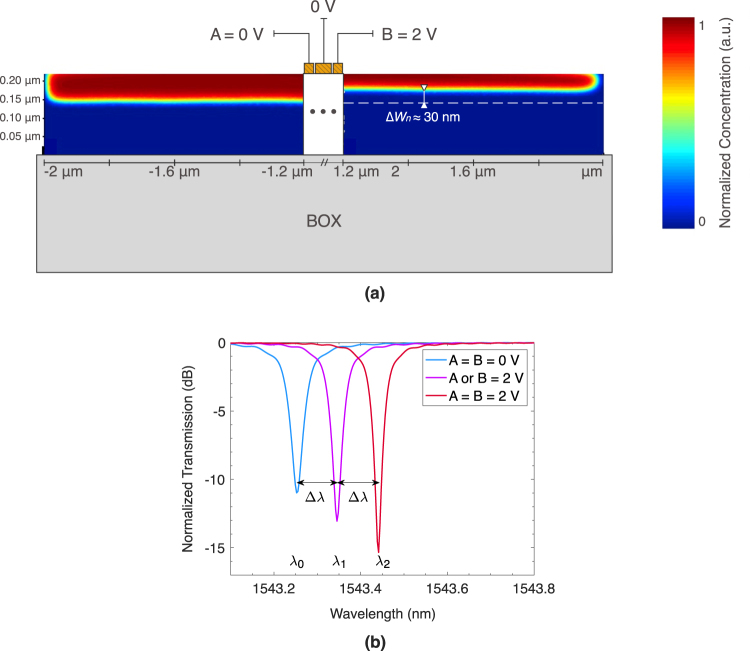
(**a**) FEM simulation visualizing the concentration of free electrons in the left- and right-side diodes operating at 0 V and −2 V biases, respectively (concentration of free holes not shown). The right-side diode shows a 30 nm larger electron depletion width Δ*W*_*n*_, while the left-side diode remains undisturbed. (**b**) FEM–FDTD simulations showing the selective through-port transmission spectrum of the disk under zero, one (A or B), and two (A and B) 2 V controls.

In our simulated analysis, a 3 μm thick layer of buried oxide sits beneath the 0.22 μm thick silicon disk and bus waveguides. We choose a disk radius of 2 μm for high-density integration. The bus waveguides are 0.4 μm wide and are spaced 0.16 μm away from the disk. The n-doped region extends to the outer edge of the disk, has a concentration of 3 × 10^17^ cm^−3^, and a depth of 0.11 μm. Likewise, the p-doped region underneath has a concentration of 2 × 10^17^ cm^−3^ and a height of 0.11 μm. To isolate the two diodes, a 0.6 μm wide region in the middle of the disk is left undoped. Underneath the grounded metal contact is a highly doped p+ region, with a concentration of 2 × 10^19^ cm^−3^, an upper-half radius of 0.6 μm, and a lower-half radius of 1.2 μm. Underneath the active metal contacts are highly doped n+ regions, with concentrations of 3 × 10^19^ cm^−3^ and outer radii of 1.2 μm. These highly doped regions are added to provide higher conductivity with the metal. In Fig. 2a, a finite-element method (FEM) simulation using Lumerical’s DEVICE software^42^ shows the two diodes working independently of each other, where the concentration of free electrons (in this example) spreads by Δ*W*_*n*_ = 30 nm for the reverse-biased right diode (−2V), while remaining confined in the center for the unbiased left diode. These simulations are then imported into Lumerical’s finite-difference time domain (FDTD)^43^ optical solver to demonstrate the resonance-shifting behavior of the switch, as shown in Fig. 2b. Note that this disk has an unbiased *Q* of approximately 31,000, with *FWHM* ≈ 50 pm, and ${\rm{\Delta }}\lambda \ll FSR$. One 2 V input shifts the resonance peak by Δ*λ* = 90 pm (45 pm/V), with a modulation depth of 12.6 dB, and an extinction ratio (ER) of 13 dB. With both inputs on, an additional 90 pm shift is observed, with a modulation depth of 15 dB, and an ER of 15.3 dB.

The switching energy per diode is defined as *E* = *CV* ^2^, where *V* is the applied bias, and *C* is the capacitance of the pn junction. Given that the probability of the 0-to-1 switching case is 1/4, the energy consumed per bit is defined as *E*/*bit* = *CV* ^2^/4. Our FEM simulations show a capacitance of 12 fF per diode, resulting in 12 fJ/bit dynamic switching operation. This compares well to the experimentally verified microdisk modulators of 3^44^ and 0.9 fJ/bit operation^24^. Note that both are examples of how a better junction-to-mode overlap leads to an even higher switching efficiency. The total switching delay is defined as *t*_*switch*_ = *τ*_*RC*_ + *τ*_*photon*_, where *τ*_*RC*_ is the *RC* constant of the contacts, and *τ*_*photon*_ = *λQ*/2*πc* is the photon lifetime in the disk cavity. Given the *Q* factor of the disk in our simulations, we expect *τ*_*photon*_ = 25 ps. Because this is generally much longer than the *RC* constant of the contact points, we consider the switching delay to be primarily limited by the photon lifetime, resulting in a 28 Gbps maximum NRZ bit rate. Note that while reducing *Q* (increasing peak width) reduces delay, a corresponding increase in voltage is required to maintain the same modulation depth. This introduces a significant speed–power tradeoff. A better path of innovation comes with a more efficient use of each disk (this work), by increasing switching efficiencies (Δ*λ*/V) through better junction-to-mode overlapping, or by improved etching and lithography to further reduce the minimum radii of fabricated disks. Smaller devices lead to increased computational throughput per chip and decreased total capacitance and dynamic energy consumption.

Note that carrier-based switching transfers heat from the electrical contacts to the disk, adding a significant albeit slow redshift in the transmission spectrum. High-*Q*, high-sensitivity devices such as this one are especially prone to errors from internal and external sources of heating and cooling. Therefore, integrated thermal controllers may be needed for low-error operation. These have been reported to only add low femtojoule levels of energy consumption per bit^6,24,26^.

### Logic cell

**Figure 3:**
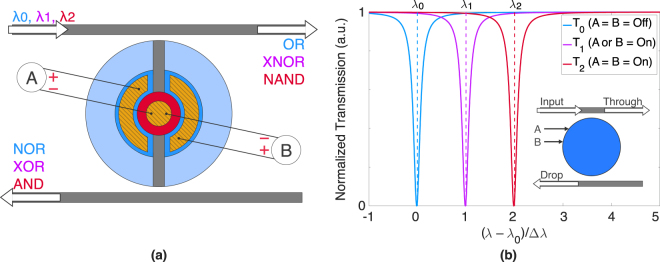
(**a**) Setup of the fundamental logic cell. Three logic signals, with separated wavelengths, are inputted into the top bus and propagate to the through or drop ports as a function of the two electrical controls, A and B. (**b**) Given the electrical input combination, the through-port transmission spectrum of the cell shifts by 0, 1, or 2 Δ*λ* to bring onto resonance the NOR, XOR, and AND operations, respectively. At any given time, one drop-port operation and two through-port operations are true. Inset shows a simplified symbol of the logic cell.

The logic setup is as shown in Fig. 3. Three different wavelengths are inputted into the cell; they operate at 0, 1, and 2 Δ*λ* away from the disk’s unbiased resonance peak of interest and are used to perform the (N)OR, (N)AND, and X(N)OR operations, respectively. Note that Δ*λ* is the wavelength shift induced by a single 2 V control (on either diode). *λ*_0_ is centered 0 Δ*λ* away from the unbiased resonance peak. If neither of controls A or B are on, the signal propagates to the drop port and sets NOR high. If either or both of the controls are on, the resonance peak shifts past the signal, which propagates to the through port, setting OR high. *λ*_1_ is centered 1 Δ*λ* away from the unbiased resonance peak. If neither of the controls are on, the signal propagates to the through port and sets XNOR high. If *only* one control is on, the resonance peak shifts into alignment with the signal, which propagates to the drop port, setting XOR high. Should both controls be on, the peak overshoots the signal, which reverts back to its original, XNOR output. Lastly, *λ*_2_ is centered 2 Δ*λ* away from the unbiased resonance peak. If neither of the controls are on, the signal propagates to the through port and sets NAND high. The same result occurs for the one-control case, as the peak has not shifted far enough to bring the signal onto resonance. When both controls are on, the resonance peak shifts into alignment with the signal, which propagates to the drop port, setting AND high. Note that with XOR and AND operations occurring simultaneously, this logic cell also produces the SUM and CARRY bits of a half adder—bringing 7*X* total logical functionality per cell.

## Higher-order logic

The optical logic cell serves as a building block to higher-order computing operations. These operations generally require the output of one stage of logic to directly drive the input of the next; however, this necessitates the use of costly OE converters and adds inherent delays from its one-after-another operational structure. Instead, we use a directed logic approach, in which multiple logic cells are concatenated, and a single or multiple optical logic signals propagate from input to output according to which cells are in on or off resonant modes. All cells are switched simultaneously in this approach, giving it a significant advantage over conventional logic, as switching delays do not scale with the size of the operation. To specify each cell’s gate operation(s), their resonance peaks of interest are designed (or perhaps tuned) to operate 0, 1, or 2 Δ*λ* away from the optical logic signal(s). As a proof of example, we demonstrate how this approach is used to perform five important logic circuits in modern computers; these include *N*-bit AND/OR gates, adders, comparators, encoders, and decoders. Low-bit-count examples are shown here, with additional discussions on their bitwise expansion. With ultracompact design, simultaneous cell switching, and the use of high-performance silicon-photonic modulation schemes, these circuits show significant improvements over previous demonstrations of optical logic while introducing certain advantages over digital logic.

### N-bit AND/OR

**Figure 4:**
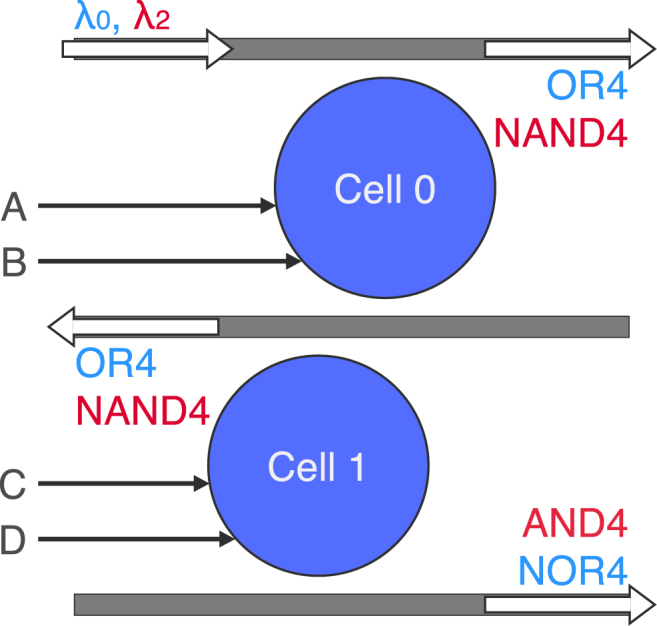
Schematic of the 4-bit AND/OR gate. Two drop-port coupled logic cells simultaneously perform AND4 and OR4 operations (and their inversions) on two separate optical logic signals.

Figure 4 shows a 4-bit AND/OR gate using two drop-port coupled logic cells. *λ*_0_ and *λ*_2_ (*λ*_0_ + 2Δ*λ*) are inputted at the top waveguide. When all four input controls (A, B, C, and D) are on, *λ*_2_ propagates down to the drop port of Cell 1 as a function of AND4; otherwise, it exits at one of Cell 0’s output ports, where it is to be filtered out or accepted as a function of NAND4. When no controls are on, *λ*_0_ propagates to the drop port of Cell 1, where it is to be filtered out or accepted as a function of NOR4. If A or B are switched on, *λ*_0_ propagates to the through port of Cell 0; if not, and C or D are switched on, it will propagate to the next drop port. Note that the waveguides shown in these figures are kept short for visual demonstration only; further routing may be needed in the fabrication layout. For larger bit counts, additional cells are coupled by their drop ports. *N*/2 disks are used for *N*-bit AND/OR operations, resulting in an ultracompact 1/4 disks per operation per bit (1/8 if NAND and NOR operations are accepted). To the best of our knowledge, the only previously demonstrated microresonator-based optical *N*-bit AND/OR gates are with reconfigurable directed logic, using one ring per operation per bit^31^.

### N-bit comparator

**Figure 5:**
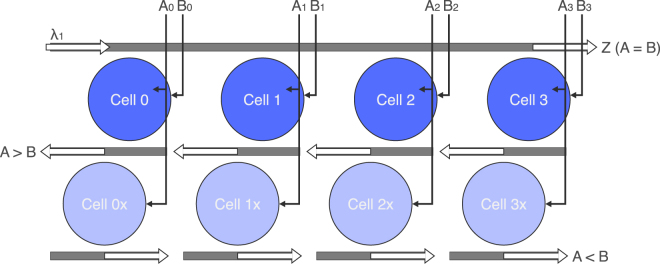
Schematic of the 4-bit comparator. Four through-port coupled logic cells determine if two 4-bit numbers are equal. Cells 0x–3x are added to determine which number is larger; they are aligned with *λ*_0_.

Figure 5 shows a 4-bit comparator using four through-port coupled logic cells. A comparator is a fundamental logic circuit, used in various stages of a microprocessor’s pipeline (decoding, arithmetic, addressing), that determines the equality of two binary numbers. If a greater/less than comparison is required, an additional four cells are drop-port coupled underneath. *λ*_1_ is inputted into Cell 0 and propagates to the through port of Cell 3 as a function of

1 $${\rm{Z}}=(\overline{{{\rm{A}}}_{3}\oplus {{\rm{B}}}_{3}})(\overline{{{\rm{A}}}_{2}\oplus {{\rm{B}}}_{2}})(\overline{{{\rm{A}}}_{1}\oplus {{\rm{B}}}_{1}})(\overline{{{\rm{A}}}_{0}\oplus {{\rm{B}}}_{0}}).$$

Recalling the operation of a single cell, *λ*_1_ operates at 1 Δ*λ* away from the resonance peak of interest and exits at the through port as a function of XNOR. Passing through multiple, serially coupled cells ANDs multiple XNOR operations together. If A_*n*_ ≠ B_*n*_, *λ*_1_ resonates with Cell *n* and propagates to the corresponding cell underneath to check which input triggered the inequality. Only one input control is necessary for this check cell—say A_*n*_—because it is centered on *λ*_1_ (red-detuned 1Δ*λ* from the standard cell). If the logic signal exits at the through port, it is because A_*n*_ switched it, and therefore A > B. Otherwise, if the signal exits at the drop port, we conclude that A < B. Note that, again, additional routing and coupling will be required to combine the multiple output ports of the greater/less than operations. This circuit presents the most compact design for an optical bitwise comparator, simply using *N* disks for an *N*-bit equality comparison, and an additional *N* disks for the greater/less than functionality. Previously reported microresonator-based bitwise comparator designs use 59 rings (plus an OE conversion) for a 4-bit operation^31^ and 17 rings for a 2-bit operation^34^.

### N-bit adder

**Figure 6:**
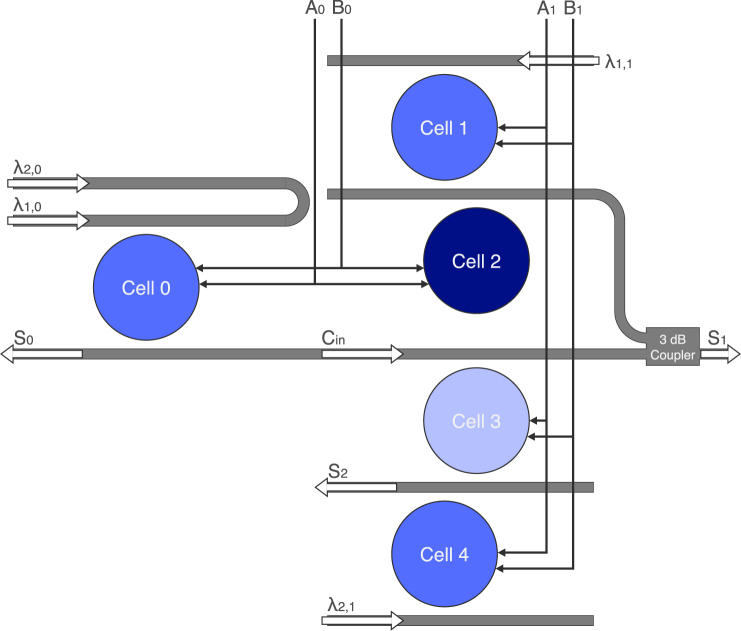
Schematic of the 2-bit optical adder. Cell 0 represents the half adder of the first stage of addition, and Cells 1–4 represent the full adder of the second stage. Cells 2 and 3 are blue- and red-detuned 1 Δ*λ* away from the standard (blue) logic cells, respectively. For *N*-bit operations, *N* − 1 full adders are connected in series, for a total of 4*N* − 3 disks.

Figure 6 shows a 2-bit optical adder. An adder is a fundamental digital circuit of a microprocessor’s arithmetic logic unit (ALU), in which every instruction of a computer is passed through. It is also used to generate memory addresses for loading and storing data. Our design combines a single-cell half adder stage and a multicell full adder stage. Each stage has two optical inputs, *λ*_1,*n*_ and *λ*_2,*n*_, and two electrical controls, A_*n*_ and B_*n*_, that work to create the SUM and CARRY outputs. *λ*_1,0_ and *λ*_2,0_ are inputted into Cell 0 to produce S_0_ and C_*in*_ as functions of A_0_ ⊕ B_0_ and A_0_B_0_, respectively. The outputs of the full adder are defined as

2 $${{\rm{S}}}_{1}\,=\,{{\rm{A}}}_{1}\oplus {{\rm{B}}}_{1}\oplus {{\rm{C}}}_{{\rm{in}}}$$

3 $${{\rm{S}}}_{2}\,=\,{{\rm{C}}}_{{\rm{out}}}{={\rm{A}}}_{1}{{\rm{B}}}_{1}+{{\rm{C}}}_{{\rm{in}}}{({\rm{A}}}_{1}\oplus {{\rm{B}}}_{1}).$$

S_1_ is set high if only one of A_1_, B_1_, or C_*in*_ are high—or if all three are. Cell 1 produces S_1_ from *λ*_1,1_ as a function of A_1_ ⊕ B_1_. Cell 2 is introduced underneath it, and blue-detuned 1 Δ*λ*, to cancel S_1_ if C_*in*_ is high (a function of A_0_B_0_). Cell 3 (red-detuned 1 Δ*λ*) cancels S_1_ as a function of A_1_ ⊕ B_1_ to round out the possible cases for equation (2), while simultaneously producing the second product of equation (3). Cell 4 works on *λ*_2,1_ as a function of A_1_B_1_ to produce the first product of equation (3). As a sum of products, Cells 3 and 4 can set S_2_ high. This presents the most compact design for an optical adder, using 4*N* − 3 disks for an *N*-bit operation. Previously reported ring-based adder designs use two rings for a half adder^35^ and 15 rings for a 2-bit adder^36^.

### N-bit encoder and decoder

**Figure 7:**
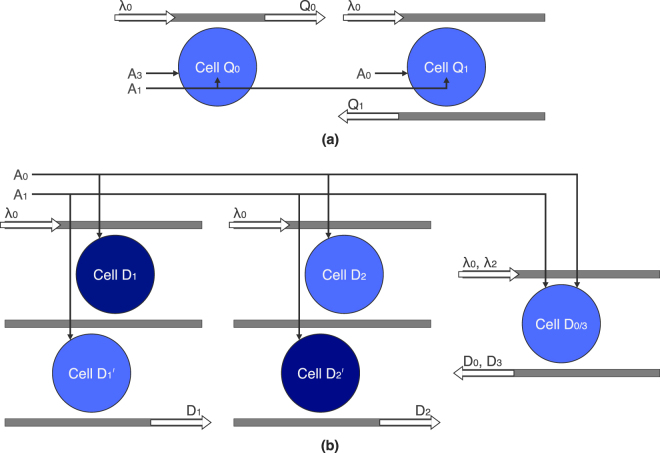
Schematics for the (**a**) 4-to-2 encoder and (**b**) the 2-to-4 decoder. Each circuit is a collection of AND and OR gates that can be arranged for a more optimized use of space in application-specific circuits. The dark blue cells are blue-detuned 1 Δ*λ* away from the standard blue cells.

Figure 7a shows a 4-bit encoder. This circuit compresses a 4-bit number into a 2-bit binary representation of itself, as shown in Table 2, for reduced-bandwidth-use data transfer. This is done by two separate suboperations,

4 $${{\rm{Q}}}_{0}{={\rm{A}}}_{1}+{{\rm{A}}}_{3}$$

5 $${{\rm{Q}}}_{1}=\overline{{{\rm{A}}}_{0}}\overline{{{\rm{A}}}_{1}}=\overline{{{\rm{A}}}_{0}+{{\rm{A}}}_{1}},$$

performed by two separate logic cells. Because these cells are not connected, they can be arranged differently for each application-specific circuit for a more optimized use of space. Q_1_ is converted from an AND operation of two inverted control signals, by De Morgan’s law, to a NOR operation of two noninverted controls. For higher-order encoding, the necessary AND and OR operations are added. For example, an 8-to-3 encoder can be reduced to three 4-bit OR gates

6 $${{\rm{Q}}}_{0}{={\rm{A}}}_{1}+{{\rm{A}}}_{3}+{{\rm{A}}}_{5}+{{\rm{A}}}_{7}$$

7 $${{\rm{Q}}}_{1}{={\rm{A}}}_{2}+{{\rm{A}}}_{3}+{{\rm{A}}}_{6}+{{\rm{A}}}_{7}$$

8 $${{\rm{Q}}}_{2}{={\rm{A}}}_{4}+{{\rm{A}}}_{5}+{{\rm{A}}}_{6}+{{\rm{A}}}_{7},$$

which require six logic cells. This is reduced to five cells, given that the A_5_ + A_6_ operation is shared amongst two of the three operations. We believe that this is the most compact design for an optical encoder, using two and five disks for the 4-to-2 and 8-to-3 encoders, respectively. The only previously reported design is an 8-to-3 priority encoder, which is inherently more complex, using 47 microresonators and an OE conversion^31^.

**Table 2:** Truth Table for a 4-to-2 Binary Encoder.

A_3_	A_2_	A_1_	A_0_	Q_1_	Q_0_
0	0	0	1	0	0
0	0	1	0	0	1
0	1	0	0	1	0
1	0	0	0	1	1
0	0	0	0	X	X

Binary decoders are also more complex in design. Figure 7b shows a 2-to-4 decoder using five logic cells. This circuit produces the original 4 bits of the 2-bit encoded number by

9 $${{\rm{D}}}_{0}=\overline{{{\rm{A}}}_{0}}\overline{{{\rm{A}}}_{1}}=\overline{{{\rm{A}}}_{0}+{{\rm{A}}}_{1}}$$

10 $${{\rm{D}}}_{1}{={\rm{A}}}_{0}\overline{{{\rm{A}}}_{1}}$$

11 $${{\rm{D}}}_{2}=\overline{{{\rm{A}}}_{0}}{{\rm{A}}}_{1}$$

12 $${{\rm{D}}}_{3}{={\rm{A}}}_{0}{{\rm{A}}}_{1}.$$

D_0_ and D_3_ are combined into logic cell D_0/3_, which performs the necessary AND and NOR operations. The remaining two operations require an inverted control signal, which cannot be done easily with our logic cell. Instead, we incorporate two drop-port coupled double disk resonators, where each switch is controlled by a single input, A_0_ or A_1_. The double disk for the D_1_ operation outputs a high bit when A_0_ is high and A_1_ is low. This is done by blue-detuning Cell D_1_ 1 Δ*λ* away from the location of the standard logic cell, while Cell ${{\rm{D}}}_{1}^{^{\prime} }$ remains the same. The double disk for the D_2_ operation is configured in a similar way, except that Cell ${{\rm{D}}}_{2}^{^{\prime} }$ is blue-detuned, and Cell D_2_ is not. To the best of our knowledge, this is the only microresonator-based optical binary decoder.

## Discussion on fabrication

The purpose of this work is to propose novel designs for higher-performance optical logic, and to showcase the predicted theoretical performance with support from literature and simulation. Fabricated prototype devices are expected to verify this operation. Undoped waveguides, microresonators, and grating or edge couplers are standard silicon-photonic components that can be easily made with common lithography (e.g., electron-beam and deep ultraviolet) and etching (e.g., reactive ion) techniques. Given the target dimensions provided in our design, these components should be fabricated within reasonable tolerances. Although, as with any integrated device, small fabrication variations across the chip will certainly have an impact on the passive performance of the device (e.g., propagation loss, resonator quality factor, and free-spectral range), but this should be acceptable. It is anticipated that the fabrication of unconventional active structures such as the vertical pn junction will require special consideration for quality control and repeatability. The process is, however, relatively straightforward. Starting with a fully etched microdisk resonator, Boron (the acceptor dopant) is implanted into the silicon (masked to not expose the passive regions of the design) at an approximated dose of 200 × 10^12^/cm^2^ and energy of 35 keV (see the Methods section for details). This forms a p+ region in the lateral center of the disk, with a radius of 0.6 μm, as shown in Fig. 1. A higher-energy step (approximately 55 keV) is taken to create another p+ region, with an outer radius of 1.2 μm and an inner radius of 0.6 μm, vertically positioned at the lower half of the disk. A lower-dosage step is taken to create the p region at the lateral edge of the disk, with an inner radius of 1.2 μm. Phosphorus (the donor dopant) is then implanted at an approximated dose of 170 ×10^12^/cm^2^ and energy of 45 keV to create a pn+ junction vertically centered in the disk. Lastly, a lower-dosage n implant is used to create the pn junction at the lateral edge of the disk. All doses, energies, and dimensions are well within the reasonable range of capabilities for current ion implantation systems^45^. Nonuniformities in doping may, however, cause mismatched concentrations and junction depths between each diode, resulting in imbalanced switching. For example, in off-resonance to on-resonance operations, the mismatch may cause a misalignment between the resonance peak and the optical signal, resulting in lower modulation depths and higher bit error rates. To account for this, the circuit may be set to operate at a lower speed; or, the applied voltage may be tuned to calibrate the device to its optimal operating point. However, this is likely unnecessary, as modern ion implantation systems regularly achieve doping uniformities better than 1.5%^45^. After doping, a blanket layer of oxide is deposited by plasma-enhanced chemical-vapor deposition, followed by the etching of vias through the oxide for the deposition of metal contacts. An optional microheater may be deposited on top of the oxide. Note that the top oxide cladding also isolates the metal from the outer edge of the disk to avoid the absorption of the optical mode. We believe that this fabrication process is feasible, given the currently available technology and the successful demonstrations of doping and metalization of silicon microresonators similar to our proposed structure, in literature^24,41,44^, with reasonable quality control and repeatability.

## Conclusion

We have proposed a high-throughput CMOS-compatible optoelectronic directed logic architecture that uses ultracompact vertical pn junction microdisk switches to achieve a higher Δ*λ*/V than conventional microring switches; two electrical controls per switch to (at least) halve the size of logic operations; and wavelength-division multiplexing to perform (N)OR, (N)AND, and X(N)OR operations simultaneously on a single switch. These basic gates are expanded on to create the most compact CMOS-compatible designs for *N*-bit logic gates, adders, comparators, encoders, and decoders. By using the fewest amount of (high-performance) switches per logic operation, this architecture can achieve the highest throughput and lowest energy consumption of all previously reported optical logic. Performance can be further optimized by reducing disk radii (resulting in reduced area and capacitance/energy) and enhancing junction-to-mode overlap for ultralow-voltage operation.

## Methods

### Simulations

Using Lumerical DEVICE and FDTD software, the optoelectronic behavior of our proposed logic cell was simulated. A structure was first made in DEVICE using the included material models and primitive structures in an isothermal steady-state solver. The p and n regions have constant doping profiles; their boundaries, depths, and concentrations were defined using custom MATLAB code. The three electrodes use the aluminum material model. The anode was grounded, and the two cathodes were swept between ground and 2. Junction capacitance was calculated as a function of the swept cathode voltage (per diode) by *C*_*i*_ = (*Q*_*i*_ − *Q*_*i* − 1_)/(*V*_*i*_ − *V*_*i* − 1_). The 3D charge profile was then imported into the FDTD model, which used the same materials and structures. Three broadband FDTD simulations were run: no biased diodes, one reverse-biased diode, and two reverse-biased diodes. A short pulse of the fundamental transverse electric mode was used in each run to solve for the transmission spectra.

### Ion implantation calculations

Rough calculations were made to estimate the required dosages and implant energies for doping, and to confirm that they are within range of commercially available ion implantation systems. Given the required dopant ranges, *R*_*n*_ and *R*_*p*_, and concentrations, *N*_*n*_ and *N*_*p*_, implantation dosage is found by *Q* = *N* $\sqrt{2\pi }$Δ*R*, where Δ*R* is the straggle of the doping profile. Range and straggle values are both functions of implantation energy, and are found in the experimental data for common acceptor and donor atoms implanted into silicon^46^.

### Data availability

All simulated data are included in this manuscript.
